# Incorporating a gender perspective into the development of clinical guidelines: a training course for guideline developers

**DOI:** 10.1186/1748-5908-2-35

**Published:** 2007-11-12

**Authors:** Debby G Keuken, Joke A Haafkens, Marian J Hellema, Jako S Burgers, Clara J Moerman

**Affiliations:** 1 Department of General Practice, Division of Clinical Methods and Public Health, Academic Medical Center-University of Amsterdam, Meibergdreef 15, 1105 AZ Amsterdam, The Netherlands; 2 Dutch Institute for Healthcare Improvement CBO, Churchilllaan 11, 3527 GV Utrecht, The Netherlands

## Abstract

**Background:**

Dutch guideline-developing organizations do not focus systematically on differences between men and women when developing guidelines, even though there is increasing evidence that being male or female may have an effect on health and health outcomes. In collaboration with two prominent Dutch guideline-developing organizations, we designed a training course to encourage systematic attention to sex differences in guideline development procedures.

**Methods:**

The course is targeted towards guideline developers. Its aims are to improve awareness concerning the relevance of considering sex differences in the guideline development process, as well as the competence and skills necessary for putting this into practice. The design and teaching methods of the course are based on adult learning styles and principles of changing provider behaviour. It was adjusted to the working methods of guideline organizations. The course was taught to, and evaluated by, a group of staff members from two guideline organizations in the Netherlands.

**Results:**

The course consists of five modules, each of which corresponds to a key step in the guideline development process. The participants rated the training course positively on content, programme, and trainers. Their written comments suggest that the course met its objectives.

**Conclusion:**

The training course is the first to address sex differences in guideline development. Results from the pilot test suggest that the course achieved its objectives. Because its modules and teaching methods of the course are widely transferable, the course could be useful for many organizations that are involved in developing guidelines. Follow-up studies are needed to assess the long-term effect of the course on the actions of guideline developers and its utility in other settings.

## Background

Clinical-practice guidelines are important tools for improving patient care [1]. They provide recommendations concerning optimal strategies for prevention, diagnosis, and treatment of specific clinical conditions [2,3]. They are usually developed under the auspices of a guideline organization by a group of experts (which sometimes includes patients) representing a user group. Guideline organizations in many countries use a similar standard methodology for developing guidelines, including the following stages: formulation of key questions; formulation of a search strategy for locating relevant literature; critical appraisal of selected literature; and phrasing of recommendations for clinical practice [4-7]. Quality criteria for the development of guidelines are formulated in the Appraisal of Guidelines, REsearch and Evaluation (AGREE) Instrument, which was designed by an international group of researchers and experts in guideline development [8,9]. One criterion involves the target population of the guideline, which should be specifically described. The AGREE instrument mentions gender as one of the items that may be considered in such descriptions.

In the past, women were often underrepresented among the subjects participating in clinical health research [10], based on the belief that males and females have the same biology, except with regard to the reproductive system. In the 1980s, scientists and women's health activists began to express concerns about this approach, as it could hamper an accurate understanding of the impact of biological sex factors or socially constructed gender factors on health and disease. Impeding this type of understanding could lead to less appropriate health care services for both sexes [10]. Health research funding organizations in several countries responded to this situation by adapting their policies. For example, the US National Institutes of Health have required that men, women, and minorities should be adequately represented in clinical studies since 1993 [11]. In recent years, the number of published studies addressing sex and gender differences in aetiology, diagnosis, treatment, and prevention has increased considerably [12-17].

If quality of care for both women and men is to be improved, it is essential that the new body of evidence concerning sex and gender differences be taken into consideration when developing clinical guidelines. This conforms to the Global Platform of Action, which was adopted at the Fourth World Conference on Women in Beijing in 1995 and which is now being implemented through legislation in many countries across the world [18]. Article 105 of the Treaty states the following: "In addressing inequalities in health status and unequal access to and inadequate health-care services for women and men, governments and other actors should promote an active and visible policy of mainstreaming a gender perspective in all policies and programs, so that, before decisions are taken, an analysis is made for women and men, respectively [19]."

In the Netherlands, two guideline organizations have longstanding experience with guideline development. The Dutch Institute for Healthcare Improvement (CBO) has been developing multidisciplinary guidelines since 1981, and the Dutch College of General Practitioners (NHG) has been developing guidelines for general practitioners since 1989 [20,21]. Both organizations use an internationally accepted methodology for developing guidelines; this methodology is reflected in the handbooks they have published [7,22].

A previous study, which examined the work of seven guideline working groups of the NHG and the CBO, revealed that these groups had paid little or no systematic attention to potentially relevant evidence on sex- and gender-related factors. This was reflected in the working methods that they used in the various stages of the guideline development process and in the final content of the guidelines [23]. The study suggested several barriers to the systematic inclusion of evidence on sex-related and gender-related factors in the process of guideline development. These barriers are as follows:

1. The working groups were critical of the extensive evaluation of specific patient characteristics, as they aimed to develop recommendations for the general patient population.

2. The working groups lacked awareness that attention to sex-related or gender-related factors might improve the quality of guidelines.

3. The working groups also lacked competence regarding the identification and systematic evaluation of evidence on sex and gender differences, and the CBO and NHG handbooks for guideline developers lacked any comprehensive set of instructions on how to do this [23].

Based on the results of this study, both organizations decided to collaborate in a quality-improvement initiative to facilitate increased systematic attention to sex and gender differences during the guideline development process.

As an initial first step towards this end, we provided written recommendations for focusing on sex-specific evidence in the guideline development process [23], some of which were included in a new handbook for guideline developers published by CBO and NHG [7,24]. Evidence from systematic reviews of professional behaviour change, however, has shown that passive dissemination strategies (*e.g*., written information) are largely ineffective if they are not accompanied by more active approaches involving the target groups themselves (*e.g*., interactive educational approaches) [25,26]. The latter approaches are likely to be effective if they are used to challenge negative attitudes of professionals or to teach new skills [26].

For this reason, we designed a training course entitled 'Attention to sex differences in guideline development' for the staff members of CBO and NHG, who are charged with the task of providing methodological support to guideline working groups. This paper describes the course.

English language scientific literature commonly makes a theoretical distinction between the concepts of 'sex' and 'gender'. The term 'sex' refers to biological and physiological characteristics that define men and women, while the term 'gender' refers to social characteristics that society attributes to the sexes. For example, gender affects the kind of health risks that men and women run and the type of health behaviours that they adopt or display [27]. As is the case with many other languages, Dutch does not have two separate words for the concepts of sex and gender [28]. In this article and in the description of the course, we therefore used the term 'sex differences' to designate differences between men and women. Details about the biological or social nature of these differences are provided only if they are relevant.

## Methods

### Context

The training course was developed by a psychologist and a librarian/trainer, two MD/epidemiologists and a social scientist, in close collaboration with the directors of the guideline development programmes of CBO and NHG. This group included experts in sex, gender, and health issues and in the methodology of guideline development.

### Aims

The aim of the course is to facilitate the consideration and inclusion of relevant information on sex differences in the process of developing clinical guidelines. Based on the potential barriers identified in our earlier project, its specific aims are as follows:

1. To raise awareness concerning the relevance of attention to sex differences for clinical guidelines

2. To develop necessary competence (knowledge and skills) in the systematic consideration of sex differences in all steps of the guideline development process

3. To provide practical tools for facilitating the consideration of gender issues.

### Target groups

The course is designed for staff members of guideline organizations, guideline developers and teachers with prior experience in the principles of evidence-based medicine and evidence-based guideline development.

### Objectives

By the end of the course, participants should have:

1. Greater understanding of why attention to sex differences may be relevant in guideline development

2. Skills for determining whether sex differences are relevant to the topic of a guideline and for phrasing sex-specific key questions

3. Sex-specific search terms for locating literature in Medline, Embase and PsycInfo

4. Information about other relevant sources for sex-specific information

5. Practical experience in focus on sex differences when appraising studies

6. Practical experience in the critical reading of reported subgroup analyses

7. Examples of various options for describing sex-specific information in guidelines.

### Training methods

Because the target group of the course consists of adults with substantial knowledge and experience in guideline development, the instructional methods of the course were designed to have the most impact and relevance to adult learners. Adult learners want to know why they need to learn something; they want to acquire knowledge and skills that adds to their experience; they learn from the experience of other learners, and they learn best when the topic is of immediate value to their practice [29]. Because the experience of the course participants plays a large role in adult learning, learner-centered methods are more suitable than are teacher-centered methods [30,31]. Adult learning requires the trainer to adopt a moderating style and to use flexible methods that facilitate reflection and more specific learning [29].

### Pilot testing of the course

The course includes a collective evaluation of what has been learned. For the purpose of our project, participants were also asked to complete questionnaires in order to evaluate the programme, trainers and content of the course along a ten-point scale (with 1 indicating very poor and 10 indicating very good) and to indicate three learning points. These learning points were coded according to the seven objectives of the course, using the MAXqda2 program for data analysis.

## Results

### Design

The course consists of five modules: an introductory module that discusses the purpose of the course and four modules that correspond to the various stages in the process of guideline development (assessment of the scope of and phrasing key questions for a guideline, searching literature, critical appraisal of evidence, and phrasing of recommendations). The topics of these modules are quite similar to those addressed in the introductory course on guideline development that is offered by the CBO [22]. This format was chosen because educational approaches are most likely to facilitate behaviour change in professionals when they are tailored to the attitudes, knowledge, skills, habits, and routines of the target group [26]. Moreover, the selected format also facilitates the incorporation of this course or parts of it into the regular training programmes of the guideline organizations.

The main role of the trainers in our course is to facilitate adult learning. Corresponding to the principles of adult learning, we have chosen a combination of training techniques, tailored to the following specific objectives:

1. Group and subgroup discussions to facilitate reflection on the participants' own attitudes regarding the relevance of sex differences to guideline development, and for the purpose of developing ideas and concepts

2. Short instructions by the trainer, assignments, as well as group and subgroup reflection about the assignments to facilitate more specific learning about incorporating attention to sex differences into the various stages of guideline development.

Educational materials consisted of a course manual for participants and trainers, including a set of tools, a PowerPoint presentation to guide the course and introduce each new topic, and a flipchart. The course was designed to be taught in two afternoon sessions of two to three hours each. The participants were asked to review the course materials in advance of each session.

### Content

The training course consists of five modules (see Additional File 1: AF1 Training course.pdf for a detailed description of the modules of the training course).

Module one is the introduction to the training course; its purpose is to increase awareness of the potential relevance of integrating attention to sex differences into the guideline development process. The module consists of two parts. It starts with an icebreaker to assess and discuss the opinions of the training-course participants regarding the potential relevance of paying attention to sex differences. The second part consists of a brief lecture by the trainer, explaining the scope, aims, and objectives of the training course, followed by an introduction about a number of key concepts. The module ends with a group discussion, facilitated by the trainer, to elicit reflection on the course programme and the key concepts.

Each subsequent module corresponds to one of key steps in the guideline development method:

Module two addresses the following questions: How can potentially relevant sex differences be assessed with respect to the topic of the guideline? Which type of key questions would allow sufficient attention to these differences? The module starts with a group discussion, in which the participants are asked to provide examples of questions that could be used to assess sex differences related to the general topics that are addressed in guidelines (*e.g*. epidemiology, aetiology, diagnosis, pharmacotherapy). They are subsequently provided with a tool containing examples of sex-specific questions and research evidence according to the main topic areas of clinical guidelines. Participants are then asked to complete two assignments using this tool. First, they are asked to reflect on whether any of these questions would be helpful in formulating potentially relevant sex-specific key questions for a guideline on which they have worked. Second, they are asked to explore whether and how key questions formulated in an existing guideline should be rephrased into sex-specific questions. Both assignments are completed by subgroups of two participants. The module concludes with a short plenary discussion about the results of the assignments.

Module three addresses the following question: Which literature search strategies may allow the identification of potentially relevant literature on sex differences? This module provides written tools for locating published studies on sex differences. Examples of relevant databases are provided, along with the specific search terms that are relevant for the most commonly used bibliographic databases in biomedicine (*i.e*. Medline, Embase, PsycInfo). The trainer provides examples of how the tools can be used. If internet facilities are available, a training assignment may be given that involves using the tools.

Module four addresses the following question: How can we assess whether the retrieved studies provide relevant information about sex differences? This module starts with an introduction by the trainer of a written tool that provides examples of specific questions regarding sex differences that may be relevant for the critical appraisal of research publications. Participants are divided into subgroups and given an assignment in which they must apply the tool by assessing the abstracts of selected publications. The results of the assignment are discussed in a plenary session. The participants subsequently receive plenary instruction about a written tool for assessing sex-specific information within articles. Specific attention is paid to assessing the quality of the statistical methods used in studies (subgroup analysis). Each participant then receives an individual assignment to apply this tool to the methodology sections of a number of selected publications. The module concludes with a plenary assessment and group discussion of the results of this assignment.

Module five addresses the following question: How can information on sex differences be integrated into the final guideline document? The trainer introduces this topic by providing examples of various ways of presenting relevant information on sex differences in guidelines. A group discussion follows to facilitate reflection on whether and how the participants could use such examples in their daily practice.

### Pilot testing of the course

Draft versions of the course were tested and discussed with three experts in guideline development and teaching. The training course was given in two sessions in March and April 2005 at the offices of the CBO and the NHG. The first session covered Modules one through three; the second session covered the remaining two modules. An interval of two weeks was included between the two sessions, during which the participants were encouraged to complete assignments or engage in reflection.

Fourteen staff members participated in the pilot test. Twelve staff members participated the first session, and eleven staff members attended the second session. Nine of these participants attended both sessions. All participants completed the questionnaires, with the exception of one participant, who had to leave the last meeting early.

The content, programme and trainers were evaluated after the first session (n = 12). Course ratings varied from 7 to 9, inclusive, with means of 7.5, 7.8 and 8, respectively.

Learning points were evaluated after each session. In answer to the question of what they had learned from the course, participants noted forty-five statements on 22 evaluation forms. Table 1 shows how the statements were coded. Next to the seven objectives of the course, an additional theme emerged during the analysis: 'general methodology for integrating attention to sex differences in guidelines'. This theme referred to the practical applicability of the sex-specific methodology learned in the training course. Fourteen of the statements referred to this theme. Five statements referred to other topics that neither referred to one of the other objectives/themes nor raised another new theme. One of the forty-five statements had a double coding, and one statement had a quadruple coding.

**Table 1:** Pilot test: Learning points of the course

**Course objectives**	Module*	**Number of statements**	**Quote**
1. Greater understanding of why attention to sex differences may be relevant in guideline development	1	14	*"Gender does matter, but other forms of diversity can be relevant as well. It is right to consider these matters throughout the guideline development process"*.
2. Skills for determining whether sex differences are relevant to the topic of a guideline and for phrasing sex-specific key questions	2	4	With reference to the tool: *"assessing with the help of criteria rather than 'unquestioningly doubling' key questions by gender"*.
3. Sex-specific search terms for locating literature in Medline, Embase and PsycInfo	3	4	*"It is good to know that there are sex-specific search filters"*.
4. Information about other relevant sources for sex-specific information	3	1	*"sources and knowledge"*
5. Practical experience in how to focus on sex differences when appraising studies	4	4	*"sex-specific checklist for literature assessment"*
6. Practical experience in the critical reading of reported subgroup analyses	4	10	*"very clear explanation of subgroup analysis"*
7. Examples of various options for describing sex-specific information in guidelines	5	2	*"different ways of including it in the guideline"*
**Other codes**			
General methodology for integrating attention to sex differences in guidelines	1–5	14	*"specific practical leads with which to work"*
Other	1–5	5	*"Discussion about the homework: the correct answers that were provided were too strict. It has to be put into perspective that the parts that I assessed as right were not obviously false"*.

**Total**		58	

* Modules 1 to 3 were covered in the first session; the second session covered Modules 4 and 5. Statements from 12 evaluations of the first session and 10 evaluations of the second session.

Eleven of the statements expressed that participants had learned skills other than or in addition to those related to a sex-specific approach to guideline development. Nine of these statements referred specifically to the utility of the module on the critical reading of subgroup analysis.

## Discussion

This paper describes an educational course that was designed to help guideline development organizations consider sex differences in the design of clinical practice guidelines. The course targeted experts in evidence-based guideline development. Educational theories emphasize the importance of tailoring educational interventions to the needs and specific characteristics of the target groups [26]. For this reason, the format of the course (five modules) follows the common stages in the process of guideline development [4-7], and it uses a variety of educational strategies that have proven useful in supporting adult learning [29-31]. A previous study in two Dutch guideline organizations suggests that the barriers that impede guideline developers from considering sex differences in guideline development include lack of awareness and lack of the knowledge and skills that are necessary to implement such consideration [23]. Therefore, the objectives, content and educational strategies of the course were aimed at raising awareness and stimulating the development of practical skills.

Because we did not assess the long-term effects of the training course, we do not know whether the participants' behaviour has changed. Nevertheless, the statements on what the participants had learned from the course reflect themes that are considered important conditions for behavioural change among professionals in the literature on quality improvement in health care [26]. The results of the evaluation of the pilot test suggest that the current design of the training course is successful in achieving its objectives. We are currently conducting a follow-up study to assess whether and how the participants are implementing the knowledge and skills that they acquired in the course in their guideline development practice. The findings of this study will provide information concerning whether the training course needs further improvements.

## Conclusion

Clinical guidelines are often used as tools for quality improvement in clinical practice. According to international treaties on gender equality, guideline organizations are charged with the task of ensuring that evidence on sex differences is considered in guidelines. Active educational approaches seem to be an effective way to raise awareness of innovations among professionals. Our training course is the first to address sex differences in guideline development. Because the modules and the teaching methods are widely transferable, the training course could be useful for many organizations that are active in guideline development.

## Competing interests

The author(s) declare that they have no competing interests.

## Authors' contributions

JH and DK made a blueprint of the training course. JH, CM, MH developed the training course, in dialogue with JB. The training course was given by JH and MH. DK analysed the data, in dialogue with JH. DK drafted the manuscript. JH helped to draft concepts of the manuscript. CM, MH and JB helped to draft a later concept of the manuscript. All authors read and approved the final manuscript.

## Supplementary Material

Additional File 1 Description of the modules of the training course 'Attention to sex differences in guideline development'. This file provides a detailed description of the training course. Click here for file

## References

[B1] Grol R, Wensing M, Eccles M, Grol R, Wensing M and Eccles M (2005). Improving patient care The implementation of change in clinical practice.

[B2] Shekelle PG, Woolf SH, Eccles M, Grimshaw J (1999). Clinical guidelines: developing guidelines. BMJ.

[B3] Field MJ and Lohr KN, Institute of Medicine.Committee on Clinical Practice Guidelines. (1992). Guidelines for clinical practice from development to use.

[B4] (2004). Sign 50: A guideline developers' handbook.. http://www.sign.ac.uk/guidelines/fulltext/50/index.html.

[B5] Burgers JS, Grol R, Klazinga NS, Makela M, Zaat J (2003). Towards evidence-based clinical practice: an international survey of 18 clinical guideline programs. Int J Qual Health Care.

[B6] (2007). Handbook for the preparation of explicit evidence-based clinical practice guidelines. New Zealand Guidelines Group.. http://www.nzgg.org.nz.

[B7] (2007). Evidence-based Richtlijnontwikkeling. Handleiding voor werkgroepleden.Kwaliteitsinstituut voor de Gezondheidszorg CBO.. http://www.cbo.nl/product/richtlijnen/handleiding_ebro/handl_totaal.pdf?.

[B8] Collaboration AGREE (2003). Development and validation of an international appraisal instrument for assessing the quality of clinical practice guidelines: the AGREE project. Qual Saf Health Care.

[B9] (2004). AGREE instrument.. http://www.agreecollaboration.org.

[B10] Mastroianni AC, Faden RR, Federman DD, (Institute of Medicine.Committee on the Ethical and Legal Issues Relating to the Inclusion of Women in Clinical Studies) (1994). Women and health research;ethical and legal issues of including women in clinical studies.

[B11] National Institutes of Health Revitalization Act of 1993 (public law 103-43), 107, Stat 22 (Codified at 42 U.S.C. 289.a-1) June 10, 1993, at 486 (d) (4) (D).

[B12] Bekker MHJ (2003). Investigating gender within health research is more than sex disaggregation of data: a Multi-Facet Gender and Health Model.. Psych Health Med.

[B13] Bird CE, Rieker PP (1999). Gender matters: an integrated model for understanding men's and women's health. Soc Sci Med.

[B14] Pinn VW (2003). Expanding the frontiers of women's health research--US style. Med J Aust.

[B15] Ridker PM, Cook NR, Lee IM, Gordon D, Gaziano JM, Manson JE, Hennekens CH, Buring JE (2005). A randomized trial of low-dose aspirin in the primary prevention of cardiovascular disease in women. N Engl J Med.

[B16] Annandale E, Hunt K (2000). Gender Inequalities in Health.

[B17] Wizemann TM and Pardu ML, Institute of Medicine.Committee on Understanding the Biology of Sex and Gender Differences (2001). Exploring the biologic contributions to human health: Does sex matter?.

[B18] (2005). Review of the implementation of the Beijing Platform for Action and the outcome documents of the special session of the General Assembly entitled "Women United Nations.Commission on the Status of Women 2000: gender equality, development and peace for the twenty-first century". Report of the Secretary-General. United Nations.. http://www.un.org/womenwatch/daw/csw/csw49/documents.html.

[B19] (1995). Report of the Fourth World Conference on Women.United Nations.. http://www.un.org/esa/gopher-data/conf/fwcw/off/a--20.en.

[B20] (2007). Kwaliteitsinstituut voor Gezondheidszorg CBO.. http://www.cbo.nl.

[B21] (2007). Nederlands Huisartsen Genootschap.. http://nhg.artsennet.nl/.

[B22] van Everdingen JJE, Burgers JS, Assendelft WJJ, Swinkels JA, van Barneveld TA, van de Klundert JLM (2004). Evidence-based richtlijnontwikkeling; een leidraad voor de praktijk.

[B23] Keuken DG, Haafkens JA, Moerman CJ, Klazinga NS, ter Riet G (2007). Attention to sex-related factors in the development of clinical practice guidelines. J Womens Health (Larchmt ).

[B24] Moerman CJ, Bruijnzeels MA, Keuken DG, Assendelft WJJ, van Everdingen JJE, Burgers JS, Assendelft WJJ, Swinkels JA, van Barneveld TA and van de Klundert JLM (2004). Sekse en etniciteit in richtlijnontwikkeling.. Evidence-based richtlijnontwikkeling; een leidraad voor de praktijk.

[B25] Grimshaw JM, Shirran L, Thomas R, Mowatt G, Fraser C, Bero L, Grilli R, Harvey E, Oxman A, O'Brien MA (2001). Changing provider behavior: an overview of systematic reviews of interventions. Med Care.

[B26] Grol R, Grimshaw J (1999). Evidence-based implementation of evidence-based medicine. Jt Comm J Qual Improv.

[B27] (2007). World Health Organization. What do we mean by "sex" and "gender"?. http://www.who.int/gender/whatisgender/en/.

[B28] Doyal L (2003). Sex and gender: the challenges for epidemiologists. Int J Health Serv.

[B29] Knowles MS, Holton EF, Swanson RA (1998). The adult learner.

[B30] Newman P, Peile E (2002). Valuing learners' experience and supporting further growth: educational models to help experienced adult learners in medicine. BMJ.

[B31] Spencer JA, Jordan RK (1999). Learner centred approaches in medical education. BMJ.

