# Supplementary material for: Incorporating a gender perspective into the development of clinical guidelines: a training course for guideline developers
Source: Implement Sci. 2007 Nov 12;2:35. doi: 10.1186/1748-5908-2-35 (PMC2204033; doi:10.1186/1748-5908-2-35)
Supplement: Additional File 1 — Description of the modules of the training course 'Attention to sex differences in guideline development'. This file provides a detailed description of the training course. [file 1748-5908-2-35-S1.pdf]

Additional file 1 - Description of the modules of the training course 'Attention to sex differences in guideline development'

Module one (Introduction): Relevance of sex differences for clinical guidelines; scope and purpose of the training course

Topic: Why could it be relevant to pay attention to sex differences in guideline development?

Icebreaker

The module opens with an icebreaker. The participants are presented with the following exercise: Please indicate how you view the relevance of paying attention to sex differences in guideline development by locating yourself at either end of the wall in this room. If you locate yourself on the left side, this means you find it very irrelevant. If you locate yourself on the right side, this means you find it very relevant. Please explain why you have chosen a particular position.

Introduction by the trainer

The trainer introduces the course as follows: The task of guideline developers is to identify and evaluate the most relevant information concerning particular topics of guidelines by systematically asking and answering a number of questions. Most guidelines thus far focus on the general patient population. It is becoming increasingly recognized, that health disparities may exist between or among groups of patients (*e.g.,* according to age, socioeconomic status, ethnicity or gender). The aim of this training course is to provide background information and practical tools that may be useful, should you want to address health disparities in guideline development. The

training course focuses on differences between men and women. In recent years, it has become increasingly acknowledged that being a man or a woman may have an important impact on individual health, as well as on the aetiology, signs, symptoms, and course of diseases. It can also affect diagnosis, prognosis, treatment, or response to diagnostics and treatment modalities. Some of these effects are related to the fact that men and women have different biological characteristics that are caused by genetic and physiological differences. In the literature, such differences are known as sex differences. These effects, however, can also be related to the fact that men and women have different social and culturally constructed characteristics, which may lead to differences in lifestyle, living conditions, norms and values. The literature refers to these types of differences as gender differences. For the sake of simplicity, this course uses the term 'sex differences' to refer to both biological and socially constructed differences.

Those who are not familiar with sex and/or gender differences in health may not know how to identify and evaluate potentially relevant information on differences between men and women. This training course is meant to facilitate this task. It consists of a number of modules that correspond to the steps that are commonly used in guideline development. For each step, we discuss ways in which to address differences between men and women (modules two through five).

By the end of the course, participants should have:

1. Greater understanding of why attention to sex differences may be relevant in guideline development
2. Skills for determining whether sex differences are relevant to the topic of a guideline and for phrasing sex-specific key questions

3. Sex-specific search terms for locating literature in Medline, Embase and PsycInfo
4. Information about other relevant sources for sex-specific information
5. Practical experience in focus on sex differences when appraising studies
6. Practical experience in the critical reading of reported subgroup analyses
7. Examples of various options for describing sex-specific information in guidelines.

This module ends with a plenary discussion, facilitated by the trainer, to elicit reflection regarding the potential relevance of sex and gender factors for guideline development and the training programme.

Module 2: Assessing relevant sex differences related to the topic of the guideline and phrasing of sex-specific key questions

Topic: How can potentially relevant sex differences be assessed with respect to the topic of the guideline? What type of key questions would allow sufficient attention to these differences?

Introduction to this module: Guidelines are based on information from scientific literature. In order to locate information, it is necessary to phrase questions. This module discusses how questions can be phrased in order to find relevant information on sex differences. In the beginning stages of developing a guideline, sex differences can be considered by guideline groups as they trace bottlenecks in information regarding the topic of the guideline.

The module starts with a plenary discussion in which the participants are asked to

provide examples of questions that could be used to assess sex differences related to the general topics that are addressed in guidelines (*e.g.,* epidemiology, aetiology, diagnosis, pharmacotherapy).

To assess whether any sex differences are relevant to the topic of a given guideline, we provide a tool consisting of questions, arranged by theme, which can be addressed by the members of a guideline working group. In addition, we present matching evidence of sex differences. If any of these differences are thought to be relevant, key questions can be formulated that should subsequently be answered in the guidelines.

Tool: Examples of sex-specific questions and research evidence according to the main topic areas of clinical guidelines

Life phase

Questions regarding a person's life phase (for instance adolescence, menopause or perimenopause) may be relevant to any of the following topics.

Prevalence, epidemiology

- Do men suffer from this health problem as often as women do?
- Can specific groups be distinguished within the sexes?

Example: The prevalence of psychological depression is higher among women than it is among men (ratio 2:1) [1].

Co-morbidity

- Are there differences between the sexes with respect to co-morbidity?

Aetiology

- Could differences in sex or gender influence the cause and development of symptoms?

Risk profile

- Are men and women subject to different risk factors?
- Are definitions of the risk factors the same for men as they are for women?
- Do men and women differ in behaviours that affect the onset or development of the disease?
- Are gender-specific aspects of socialization or personal history relevant to the development of the disease? (*e.g.*, women are often more likely than men are to be victims of sexual abuse; men are more likely than women are to be war veterans.)

Example: In women, diabetes is a better predictor of cardiovascular disease than it is in men. The risk of cardiovascular disease increases in a later stage in life for women than it does for men. (Women are ten years ‘behind’ men. A 60-year-old woman has approximately the same risk that a 50-year-old man has.) The age-related risk difference disappears for women who suffer from diabetes [2].

(Patho)physiology

- Is the (patho)physiology of the disease the same for men as it is for women?

Presentation of symptoms

- Is the presentation of symptoms subject to sex or gender differences?
- Does gender influence the point at which a person seeks help in the course of the disease?

Example: Women with acute coronary syndromes (ACS) are more likely than men are to present with symptoms that are considered atypical for ACS [3].

Diagnosis

- Are the instruments that are used for a diagnosis equally valid for men and for women (with regard to specificity, sensitivity, acceptation of the examination by the patient)?
- Are the criteria that are used for diagnosing the disease in men the same as those that are used for its diagnosis in women?

Example: Exercise ECG tests for diagnosing angina pectoris are more likely to yield false positive results for women than they are for men [4].

Prognosis, course of the disease

- Is the prognosis influenced by sex?

Example: The prognosis for melanoma is worse for men than it is for women [5].

Prevention

- Could any sex-specific or gender-specific aspects be relevant to the prevention of the disease?

Treatment

- Could sex-specific or gender-specific aspects influence treatment (*i.e.,* type of treatment, patient preference for treatment, effectiveness of treatment, patient satisfaction, compliance)?
- Could sex or gender aspects influence rehabilitation?

Example: Women tend to discontinue rehabilitation treatment for heart attacks more often than men do [6].

Pharmacotherapy

- Has the medication been tested on both men and women?
- Do sex differences influence pharmacodynamics and kinetics?
- Is the therapeutic effect of the medication the same for men as it is for women?
- Is the optimal dose the same for men as it is for women?
- Are the side effects the same for men as they are for women?

Example: Alosetron is effective for women with non-constipated irritable bowel syndrome, but not for men [5].

Sex hormones

- Do sex hormones influence the development and course of symptoms?
- Do sex hormones influence the effectiveness of treatment (*i.e.,* pharmacotherapy)?
- Do sex hormones interfere with establishing a diagnosis?
- How do puberty and menopause influence clinical presentation and treatment?

Pregnancy, breastfeeding

- Does pregnancy influence the course of the disease?
- Does pregnancy influence treatment (*i.e.,* harm for mother or child)?
- What is the effect of the disease or treatment on breastfeeding?

Example: There is remission of the symptoms of rheumatoid arthritis during pregnancy [5].

Advice and education

- Are gender-specific aspects relevant for advice or education?

Expectations of patient and doctor

- Are patients' expectations with regard to their doctors and treatment gender specific?
- Is the sex of the patient relevant for a doctor?

Quality of life

- Does the disease itself or its treatment have a different effect on the quality of life for men than it does on the quality of life for women (*i.e.*, physical limitations, pain, work, relations)?
- Are there gender-specific aspects to the perception of the disease?
- Do gender-specific aspects influence the consequences of the disease or treatment for the private situation of the patient (*e.g.*, care for the patient or care that the patient provides to others)?

Communication

- Does the sex of the patient and the doctor affect the way in which they communicate about symptoms?

To practice formulating sex-specific key questions and using the tool, the participants are given two assignments, which are subsequently discussed.

Assignment one (in subgroups): Please think of a guideline on which you have

recently worked. Try to formulate sex-specific key questions for this guideline, using the formats provided in the tool as an example.

Assignment two (in subgroups): Imagine that you are member of a guideline working group on the topic of osteoporosis. The working group has formulated the following key questions as a point of departure for the guideline.

1. What is the prevalence of osteoporosis in the Netherlands?
2. What are the most clinically relevant risk factors for developing osteoporosis?
3. What is the value of diagnostic research?
4. What are meaningful prevention measurements for osteoporosis and what are the different treatments?
5. When is treatment cost-effective?

Please explore the following questions:

- Should the key question be rephrased into a sex-specific key question?
- If so, what would the sex-specific question be?

You may use the tool in your exploration.

Module 3: Locating literature on sex differences in health

Topic: Which literature-search strategies may allow the identification of potentially relevant literature on sex differences?

Introduction to this module: In order to answer the key questions for a specific guideline, guideline developers usually perform a systematic search in electronic databases that include biomedical literature. For this task, they use search terms. In this part of the training course, we provide websites and sex-specific search terms for

locating sex-specific evidence. The tool for this specific module provides information about general databases that provide information on sex or gender-related factors in health. The tool also provides information on gender-specific search terms that may be useful for locating research publications on sex differences for the electronic databases that are most commonly used in biomedicine: Medline, Embase and PsycInfo.

A plenary discussion is held, in which participants are asked about any experience they may have in searching for sex-specific information.

The trainer introduces the tool for this module.

Tool: Examples of websites

- Medlineplus is a service of the National Library of Medicine. This website provides information on women's health and men's health.

http://medlineplus.gov/

http://www.nlm.nih.gov/medlineplus/womenshealth.html

http://www.nlm.nih.gov/medlineplus/menshealth.html

- World Health Organization (WHO): The WHO actively promotes attention to sex/gender health research and policy. The website provides information on women's health and gender.

http://www.who.int/topics/womens_health/en/

http://www.who.int/topics/gender/en/

http://www.who.int/gender/en/

Tool: Examples of specific search terms

Medline (PubMed): Sex factors [MeSH], sex characteristics [MeSH], sex

differentiation [MeSH], sex [MeSH], gender differences [text word], sex differences [text word], gender [text word]

Embase (Ovid): Sex difference [subject heading], sex ratio [subject heading], gender [subject heading], sex factor [text word], gender bias [text word], gender difference [text word]

The tools are discussed in a plenary session.

Assignment: No assignment has been provided for this module. However, if computers and an internet connection are available, the participants can work with the search terms.

Module 4: Critical appraisal of sex-specific information

Topic: How can we assess whether the retrieved studies provide relevant information about sex differences?

Introduction of this module: Once research articles have been located through a systematic literature search, they must be screened for scientific and clinical relevance.

The trainer introduces the tool for this module.

Tool: Sex-specific questions for assessing evidence

The first screening involves the titles and abstracts of articles. The following can be used as an initial screening question for selecting articles on sex differences:

Do the title and abstract contain information about gender, men/women, both sexes

or a comparison between the two?

If so, the article can be assessed further by using the following questions:

- Does the research question apply to both men and women?
- What is the sex composition of the study population?
- Are both sexes sufficiently represented in the study population?
- Are differences between men and women analysed?

  If the answer to the last question is ‘no’:

    - Are the conclusions as valid for men as they are for women?

  If the answer to the last question is ‘yes’:

    - Was the subgroup analysis performed correctly?
    - Could a sex difference be of clinical relevance?

If the article contains a subgroup analysis between men and women, the following steps can be taken to obtain relevant sex-specific information:

- How was the subgroup analysis performed? Was it performed well? The participants learn how a subgroup analysis is performed statistically and how to know whether it was performed well.
- If it was not performed well, correct it.
- If it was performed well, are there any significant differences between the two sexes? (see Altman [7]).

Assignment one (in subgroups): Please use the abstract to assess the following article, which was found with help of a sex-specific search strategy, using the tool for this module:

1. Patel H, Rosengren A, Ekman I. Symptoms in acute coronary syndromes: does sex make a difference? *Am Heart J* 2004 July;148(1):27-33.

Keywords: Angina,Unstable/Arrhythmia/Article/Chest Pain/Clinical Practice/complications/diagnosis/Dizziness/Dyspnea/etiology/Experience/Fatigue/Female/Heart/Human/Male/Men/Methodology/Myocardial Infarction/Nausea/Neck Pain/nursing/Pain/Patient/Patients/Review/Sample/Sex/Sex Characteristics/Sex Difference/Support,Non-U.S.Gov't/Sweating/Sweden/Symptom/Syndrome/Vomiting/Women

Abstract: BACKGROUND: Coronary heart disease is a major problem in both men and women, but several studies have shown sex differences in symptoms of acute coronary syndromes (ACS). Some findings, however, have been disparate and inadequate, and thus a comprehensive overview of this literature would be of value. METHOD: Fifteen studies that identified symptoms of ACS for both women and men were examined through a review of the literature from 1989 to 2002. Terms used for the search included "myocardial infarction," "symptoms," "gender differences," and "acute coronary syndromes." RESULTS: Although chest pain was the most common symptom in both men and women, several differences were also noted. In all types of ACS, women had significantly more back and jaw pain, nausea and/or vomiting, dyspnea, indigestion, and palpitations. In a number of studies, which solely sampled patients with acute myocardial infarction, women demonstrated more back, jaw, and neck pain and nausea and/or vomiting, dyspnea, palpitations, indigestion, dizziness, fatigue, loss of appetite, and syncope. Men reported more chest pain and diaphoresis in the myocardial infarction sample. The designs and methodologies of the studies varied considerably. CONCLUSION: In

addition to the typical symptom of chest pain in ACS, women experience other atypical symptoms more frequently than men. Thus, there may be sex differences in the symptoms of ACS, differences that have a bearing not only on clinical practice, but also on the interpretation of available clinical studies and the design of future investigations.

Assignment two (in subgroups): Please use the following fragment of an article to assess whether the subgroup analysis was performed correctly (ten assignments and answers are available from the authors). Please use the tool for this module.

Fragment

Methods (randomized trial)

Subgroup analyses were performed for the primary efficacy variable. Subgroups were stratified by race (non-black or black), age (<65 years or ≥65 years), and sex.

Results

Overall, each subgroup experienced clinically meaningful reductions in blood pressure with both treatment regimens. The results for the subgroups comprising the largest percentage of the sample (non-blacks and those younger than 65 years of age) were similar to those for the overall population. Within treatment arms, numerical differences were seen across sex (reductions of 10.2 and 7.8 mmHg for women and men, respectively) for losartan.

The results of the assignments are discussed in a plenary session.

Module 5: Integration of sex-specific information into the guideline document

Topic: How can information on sex differences be integrated into the final

guideline document?

Introduction to this module: Once the relevant evidence has been collected, it should be written in the guideline.

While writing the guideline, there are several ways in which to pay attention to sex-specific information. In this module, several of the options that are available for presenting information on sex differences are discussed.

The trainer introduces the tool for this module.

Tool: Options for paying attention to sex differences in the composition of guideline documents

Option one: Where can information about sex differences be placed in the document?

- in the recommendations
- in the running text
- in the footnotes
- in a separate paragraph
- in separate boxes

Example (for a recommendation): ‘Women with an osteoporotic fracture of the spine or the hip under the age of 50 or men with an osteoporotic fracture of the spine or the hip under the age of 65 must be referred to a specialized care provider for further evaluation of the underlying cause’ [8].

Option two: What kind of information will be included if there is no sex-specific evidence?

- Write about sex differences only if there is evidence.
- Mention a lack of evidence.

Example (for missing information): ‘Because of lack of evidence on male subjects, it is almost impossible to make any pronouncements about the preventive value of physical exercise in men. The opinion of the working group is that the outcomes of research on women subjects can be extrapolated to men’ [8].

This tool is discussed in a plenary session.

Reference List

1. **Gender, Health and Aging. World Health Organization.** [www.who.int/gender/documents/en/Gender_Aging.pdf]

2. Kwaliteitsinstituut voor de gezondheidszorg CBO: *Herziening Richtlijn Hoge Bloeddruk. [Revised Guideline on Hypertension]*. 2000.

3. DeVon HA, Zerwic JJ: **Symptoms of acute coronary syndromes: are there gender differences? A review of the literature.** *Heart Lung* 2002, **31:**235-245.

4. **ACC/AHA 2002 Guideline Update for the Management of Patients With Chronic Stable Angina; ACC/AHA 2002 Guideline Update for the Management of Patients With Chronic Stable Angina.** [www.acc.org/qualityandscience/clinical/guidelines/stable/stable_clean.pdf]

5. Institute of Medicine: *Exploring the biologic contributions to human health: Does sex matter?* Washington, DC: National Academy Press; 2001.

6. Brezinka V, Dusseldorp E, Maes S: **Gender differences in psychosocial profile at entry into cardiac rehabilitation.** *J Cardiopulm Rehabil* 1998, **18:**445-449.

7. Altman DG, Bland JM: **Interaction revisited: the difference between two estimates.** *BMJ* 2003, **326:**219.

8. Kwaliteitsinstituut voor de gezondheidszorg CBO: *Osteoporose; Tweede herziene richtlijn. [Osteoporosis: Second, revised, guideline]*. 2002.
